# Creating a positive perception of childbirth experience: systematic review and meta-analysis of prenatal and intrapartum interventions

**DOI:** 10.1186/s12978-018-0511-x

**Published:** 2018-05-02

**Authors:** Mahshid Taheri, Amirhossien Takian, Ziba Taghizadeh, Nahid Jafari, Nasrin Sarafraz

**Affiliations:** 1 Department of Reproductive Health, School of Nursing and Midwifery, Tehran University of Medical Sciences, Tehran, Iran; 2 Department of Health Management and Economics, School of Public Health, International Campus, Tehran University of Medical Sciences, Tehran, Iran; 3 Ministry of Health and Medical Education, Tehran, Iran

**Keywords:** Childbirth experience, Psychological birth trauma, Support, Systematic review

## Abstract

**Background:**

A negative experience in childbirth is associated with chronic maternal morbidities. The aim of this systematic review and meta-analysis was to identify currently available successful interventions to create a positive perception of childbirth experience which can prevent psychological birth trauma.

**Methods:**

Randomized controlled trials of interventions in pregnancy or labour which aimed to improve childbirth experience versus usual care were identified from 1994 to September 2016. Low risk pregnant or childbearing women were chosen as the study population. PEDRO scale and Cochrane risk of bias tool were used for quality assessment. Pooled effect estimates were calculated when more than two studies had similar intervention. If it was not possible to include a study in the meta-analysis, its data were summarized narratively.

**Results:**

After screening of 7832 titles/abstracts, 20 trials including 22,800 participants from 12 countries were included. Successful strategies to create a positive perception of childbirth experience were supporting women during birth (Risk Ratio = 1.35, 95% Confidence Interval: 1.07 to 1.71), intrapartum care with minimal intervention (Risk Ratio = 1.29, 95% Confidence Interval:1.15 to 1.45) and birth preparedness and readiness for complications (Mean Difference = 3.27, 95% Confidence Interval: 0.66 to 5.88). Most of the relaxation and pain relief strategies were not successful to create a positive birth experience (Mean Difference = − 2.64, 95% Confidence Intervention: − 6.80 to 1.52).

**Conclusion:**

The most effective strategies to create a positive birth experience are supporting women during birth, intrapartum care with minimal intervention and birth preparedness. This study might be helpful in clinical approaches and designing future studies about prevention of the negative and traumatic birth experiences.

**Electronic supplementary material:**

The online version of this article (10.1186/s12978-018-0511-x) contains supplementary material, which is available to authorized users.

## Plain English summary

The negative birth experience has been common event internationally; it may create a psychological birth trauma (PBT) for women which can lead to post- traumatic stress disorder (PTSD). This study’s aim is to collect effective prenatal and intrapartum care practices to prevent negative birth experiences. To achieve this goal, an outcome-based systematic review on RCTs was conducted.

We aimed to find any intervention which can impact on the childbirth experience among low risk pregnant or childbearing women. Among 8685 search results published between 1994 and 2016, 20 unique RCTs were included.

This review categorized strategies into four main group: supporting women during labour, relaxation and pain relief during birth, intrapartum care with minimal intervention, and birth preparedness. Successful strategies in prevention of negative birth experience were presence of a trained birth companion, relaxation through massage and music, early labour assessment to minimize obstetric interventions, and being prepared for childbirth through individual birth plan.

This newly found list of successful strategies can shed light on clinical practice in order to create a positive perception of childbirth experience. We believe that emotional support programs for childbearing women should be implemented in countries’ maternal health plans. These programs can comprise a combination of successful strategies such as continuous labour support by a familiar person, reassuring physical contact using massage, and the continuity of midwifery care. Prevention of negative birth experience using these successful practices leads to the promotion of vaginal birth, high quality maternity care and the reduction of chronic psychological complications.

## Background

Childbirth is one of the most challenging psychological events in a mother’s life, as 10–34% of all childbearing women are faced with traumatic birth experiences [1, 2]. A negative experience in childbirth is associated with post-traumatic stress disorder (PTSD), disruption to interpersonal relationships, dysfunctional maternal-infancy bonding [3–5], reduction in rates of exclusive breastfeeding [6], inappropriate utilization of maternal and newborn care services [7], fear of childbirth and increased desire for an elective caesarean section in future pregnancies [8, 9].

Prevention of psychological birth trauma (PBT) has been recommended as a new area of research in Oxford meeting. The meeting concluded that there are no published studies directly aimed at preventing psychological trauma in childbirth [10]. However, trials that tried to create positive childbirth experiences may be considered as an alternative approach in the prevention of PBT [11].

Our current review of randomized controlled trials (RCTs) assesses all types of women-centered interventions designed to create a positive childbirth experience. Previous reviews did not aim to provide a comprehensive insight into the prevention strategies of negative childbirth experiences [12]. Therefore, it is evident there is a lack of recommendations within national guidelines regarding the prevention of PBT [1, 13]. In 2002 a narrative systematic review of observational studies, RCTs and systematic reviews was conducted to assess the effect of pain on women’s sense of satisfaction with childbirth experience [14]. In addition, Cochrane reviews about specific interventions, such as midwife-led care, have reported and analyzed perceptions of the childbirth experience as an outcome [15–17]. These reviews identified effective strategies for a positive childbirth experience, including continuous support for women during childbirth, midwife-led continuity model of care, and behavior of the caregivers. [14–16].

Early postpartum debriefing interventions as a prevention of psychological trauma were not found to be effective, therefore focusing on prenatal and intrapartum interventions was recommended [18]. Such interventions directly deal with pregnant or childbearing women as a target population [14]. The experience of childbirth involves various maternal feelings such as control over the birth, self-esteem, fulfillment, decision making and the sense of achievement; therefore, it is not surprising to encounter inconsistency across experience tools in different studies.

Considering the serious burden of PBT [3, 4, 6, 8, 9], identifying effective evidence-based interventions that promote positive birth experience is essential. According to our comprehensive search of available database, there is no review that provides a comprehensive list of strategies for creating a positive perception of childbirth. The classification of related evidence and identification of successful approaches will help policymakers in the planning of clinical practice. This systematic review aims to summarize the effect of prenatal and intrapartum interventions on maternal perception of the childbirth experience.

## Methods

The search process was conducted between July and September 2016. This research was ethically approved by Tehran University of Medical Sciences ethics committee (reference number IR.TUMS.VCR.REC.1395.374). Electronic databases included Embase, PubMed, Scopus, Web of Science and Cochrane Central Register for Clinical Trials; dissertations were searched in ProQuest theses database. Persian and Turkish databases, World Bank literature and Proceedings of relevant conferences were also searched. Expert researchers in this field were contacted by e-mail to inquire about unpublished or soon to-be-published RCT’s. An electronic search strategy was constructed using text words (Additional file 1).

A comprehensive systematic search was performed on literature published between 1994 and September 2016. The fourth edition of “Diagnostic and Statistical Manual of Mental Disorder” (DSM-IV, 1994) recognized that childbirth could be a traumatic event that may cause PTSD [19]. Since then, studies began to assess maternal experience with regard to psychological aspects of childbirth [20]. Relying on this logic, we selected the year 1994 to initiate the systematic review. Randomized controlled trials (cluster or individual) that met all the following criteria, were included:

- *Population*-Low risk pregnant or childbearing women; low-risk refers to a woman aged 18–35 with no diagnosis of complications such as hypertension, diabetes mellitus, cardio-vascular disease, multiple gestation and fetal growth restriction.
- *Intervention*-Any intervention in pregnancy or labour which aimed to improve the childbirth experience.
- *Control*-Usual care; routine care provided by personnel based on the clinical guidelines of maternity care unites and hospitals.
- *Outcome*-Women’s self-evaluation of their childbirth experiences.

Using a definition from a literature review [21], we defined childbirth experience as a woman’s self-assessment of long-term memories of their childbirth event. This definition reflects most of the key elements of childbirth experience such as feelings of control, fulfillment of expectations [22], confidence and participation in decision making [21]. We included studies which had measured the childbirth experience using the validated “childbirth experience questionnaire” or a direct question about overall perception of the childbirth experience. Unlike earlier reviews [14, 15] we did not include RCTs which defined childbirth experience as a satisfaction with care, satisfaction with pain relief and experience of pain. Also, PTSD following negative birth experience did not considered as an inclusion criterion. In addition, this review covers a wider range of related interventions than others and focuses on women-centered interventions designed to improve the childbirth experience.

Full text of the included study needed to be in English, Persian or Turkish (the first author is fluent in these three languages). As childbirth experience might be affected by maternal socioeconomic status [23, 24], we removed studies conducted among women belonging to a special socioeconomic situation (e.g. disadvantaged community, migrants and high society). Also, studies performed among women with diagnosed mental illness, pre-term (< 37 weeks)/post-term (> 42 weeks) pregnancies, obese mothers, and women with prolonged labours were excluded; diagnosis of prolonged labour was made when labour progress (cervical dilatation) crossed the partogram action line.

### Quality assessment and data extraction

Study inclusion, quality assessment and data extraction were conducted by two authors (M.T. and N.S) independently. A senior researcher (Z.T.) resolved disagreements by discussion. Quality assessment was based on the PEDro (Physiotherapy Evidence Database) scale and a modified Cochrane risk of bias tool for quality assessment of RCTs. The PEDro scale rated the trials’ methodological quality from 0 to 10 based on what they reported; the domains of scale are random allocation, allocation concealment, baseline similarity, blinding, measure of key outcomes from ≥85%, intention to treat analysis, between group comparison and point measures. RCTs with PEDro scores ≥6 were re-evaluated with the Cochrane tool [25]. Additional domains of the Cochrane tool (not available in PEDro) are addressing incomplete outcome data, selective reporting, and other sources of bias. It should be noted that double blinding was not possible for these kind of interventions, therefore studies without blinding were not considered as a high risk of bias [15, 16].

RCTs with low risk of bias were included in the review, without considering their results. The Cochrane Public Health data extraction template was modified to suit this review (Additional file 2). The modified version was piloted on eight random RCTs, then re-modified accordingly. The extracted information were study details, characteristics of participants, setting, characteristics of interventions, outcomes, details of methods and results, the specific details of childbirth experience (outcome definition, time points measured, psychometric properties of measurement tool, final result) and key conclusions.

### Statistical analysis

Due to the heterogeneity of interventions, a “comprehensive meta-analysis” was not appropriate [26]. However we conducted a separate analysis when two or more studies had similar interventions. Four separate meta-analyses were performed for three different types of interventions. Data of the six trials [27–32] with the same intervention (support during labour) were pooled and analyzed using the Review Manager software (RevMan5). Another analysis was conducted for two studies [33, 34] with similar intervention (early labour assessment). These two sets of data were dichotomous, so results were presented as risk ratio (RR) with 95% confidence intervals (CIs). The random-effects Mantel-Haenszel model was used when I^2^ value was greater than 30%.

Continuous data from two studies [30, 35], that applied continuous support during labour as an intervention strategy, were pooled and reported as mean difference (MD) with 95% CIs using random-effects Inverse Variance method [26]. A separate analysis was performed for two trials [36, 37] with the same intervention (water relaxation during labour). Other studies which were inappropriate for meta-analysis were summarized qualitatively. The point measures and measures of variability were recalculated or taken directly from the published reports.

## Results

**Fig. 1:**
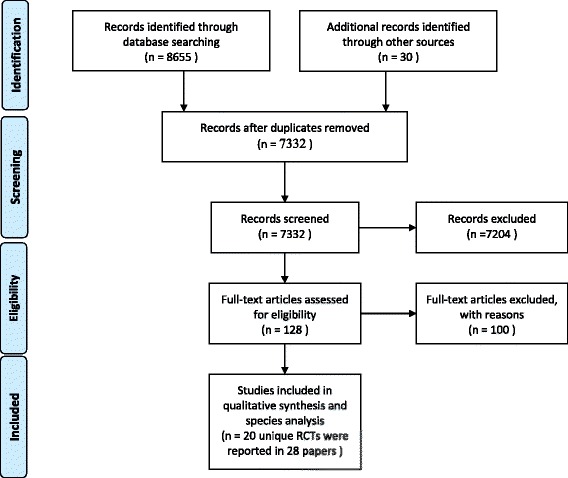
The PRISMA flow-diagram describing the screening process

In total, 8685 papers were identified in the systematic literature search. We aimed to find any intervention which can impact on the childbirth experience, so an outcome-based search strategy was required. Due to the nature of the search strategy, this large amount of papers was unavoidable. Duplicate papers were removed, and during initial assessment 7832 titles were screened by two members of the study team (M.T and N.S); 6359 non-RCT articles were excluded at this stage. Then, they screened the abstracts of 1473 papers. The trials were removed if their abstracts did not meet the inclusion criteria. Based on the abstracts, it was apparent 41 papers had relevant interventions and outcomes. However, the information presented in 87 abstracts was insufficient for making decisions about the relevance of the trial so the full texts were evaluated. The references of these 128 papers were checked to discover additional related articles. As shown in Fig. 1, 28 papers based on 20 unique trials are included in this review. Table 1 shows the characteristics of the included studies.

**Table 1:** Summary of included RCTs

Study (by grouping studies according to intervention)	Setting/Participants/Sample size/Follow up rate	Intervention	Childbirth experience measurement (definition/time points/psychometric properties)	Final results and key conclusion
Supporting women during birth				
Langer et al. 1998 Mexico	RCT in a social security hospital/ childbearing women/ *n* = 724/ 92.3% of women completed the study	The trained doula (who was hired) supported labouring woman physically and emotionally and kept her informed about labour progress.	Measured by asking a direct question with the Likert-scale responses in the immediate postpartum period.	There was no significant difference of satisfaction with the birth experience between the support group and routine care [RR = 1.01 (95% CI 0.94–1.08)]./psychological support by hired doula didn’t had a positive effect on the childbirth experience.
Gordon et al. 1999 USA	An RCT in three health maintenance organization hospitals/nulliparous pregnant women/*n* = 314/completion rate was 93%	Childbearing woman was supported by a trained doula (who was paid) during the labour and birth.	Measurement was performed by 4 questions about birth experience; questionnaire was completed at 4–6 weeks postpartum.	82.5% of women in doula Gp felt the good birth experience versus 67.4% in control Gp (*P* = .005)/The doula presence was associated with a good birth experience and coping very well with labor
Hodnett et al. 2002 USA & Canada	Multi-central RCT/ childbearing women/ *n* = 6728/ completion rate was 90%	Laboring women received continuous support by a trained nurse for a minimum 80% of the time from admission to birth.	The validated LAS (Labour agentry scale) questionnaire was used in 6–8 weeks after birth.	2740/2836 women in labour support Gp were satisfied with the birth experience versus 2646/2765 OR 1.01, 95% CI 1.00–1.02)/continuous labour support did not affect birth experience or other psychological outcomes.
Bruggmann et al. 2007 Brazil	RCT in a university hospital/ nulliparous pregnant women/*n* = 212/all of them completed the study.	Women received the labour and birth support by a chosen companion; the companion supported mother according to standardized instructions.	It was assessed by four questions asking about women feelings during labour and birth (12–24 h postpartum).	Birth satisfaction in support Gp *N* = 73 and in control Gp *N* = 60 (*P* = 0.04)/Support group had higher levels of satisfaction with the birth experience
Campbell et al. 2007 USA	An RCT in a hospital/ pregnant women/ *n* = 494/ 95% of them completed the study	Pregnant women (1 month before the birth) and their chosen companions attended two 2-h classes about the role and expectations of continuous labour support; the chosen companions provided support to women during the labour and birth.	A standardized validated questionnaire was completed by participants on average, 48 days after the birth.	Overall rating of birth experience support Gp 134 versus control Gp 68 (p < 0.001)/ labour support by traind person who selected by the mother improved maternal birth experience.
Morhason et al. 2009 Nigeria	RCT at the university hospital/ childbearing women/ *n* = 603/ follow up rate was 97%	Women received continuous support by their self-selected companion during the labour; responsibilities of the companions were explained to them and their performance were checked by attending midwives.	It was assessed by asking one closed-ended question (how would you rate your labour experience?) within 24 h following the birth.	The support Gp was 3.3 times likely to have satisfying labour experience (95% CI OR 2.15–5.04)/women who had labour support reported better labour outcomes and experiences.
Yuenyoung et al. 2012 Thailand	An RCT in regional teaching hospital/ pregnant women/ *n* = 120/ follow up rate was 95%	At a antenatal visit, the woman with her chosen close female relative participated in a 2-h preparation class to understanding of the doula’s role during labour; this close relative provided support during the labor and birth..	It was assessed by a validated LAS (Labour Agentry scale) questionnaire within 24 h after the birth.	Mean LAS score was 53.6 in the support Gp versus 47.9 in the control Gp (*P* < 0.01)/women with birth companion were more satisfied with their birth experience than other group.
Relaxation and pain relief during birth				
Rash 2000 Canada	An RCT in two birth units/childbearing women/*n* = 635/ the main outcome (epidural analgesia) were analyzed for all women but 83.3% of them completed the postpartum questionnaire.	Women were offered a bath in the labour room but they were not forced.	It was measured by the validated LAS (Labour agentry scale) questionnaire before discMge from the hospital.	Mean LAS score was 50.46 in bath Gp and 51.71 in control Gp (*P* = 0.13)/ using bath in the labour room was not recognized as an effective method to improve birth experience.
Eckert et al. 2001 Australia	RCT in a maternity tertiary referral center / Low risk Women in labor / *n* = 274/ follow up rate was 87%	Women in birth room were permitted to use a bath as little or long as they wished but water birth was not promoted.	Overall birth experience was measured using visual analog scale at 24–48 h after the birth and 8–9 months later, again.	Overall birth experience in the bath Gp was 68.74 (SD 24.31) and in the routin care was 74.62 (SD 22.08) [*P* = 0.05]/ routine care was considered more favorable experience than bath
Howell et al. 2001 UK	RCT at the Maternity department of the hospital/nulliparous labouring women/ *n* = 369/ 87% of them completed the study	Participants were given continuous epidural analgesia using 0.25% Bupivacaine during the labour.	It was assessed using two questions in the postpartum questionnaire. Measurement time points were 24–48 h, 3 months and 12 months after birth.	Maternal satisfaction with the experience of childbirth was high in both groups without significant differences (*P* < 0.40)
Kimber et al. 2008 UK	Pilot RCT in a Maternity Unit/ pregnant women booked for prenatal and birth care/ *n* = 90/ 91% of them completed the follow up questionnaire	Massage and relaxation techniques were taught to mothers and their birth companion in one session between 35 and 37 weeks by the midwife/therapist.	It was measured by the validated LAS (Labour Agentry scale) questionnaire at 6 weeks following birth	Music versus usual care MD = 6.1 (95% CI 11.9 to _0.3) and massage versus usual care MD = 6.1 (95% CI 11.6 to _0.6)/ regular massage with relaxation techniques was associated with more positive perception of labour and sense of control
Cyna. 2011 Australia	RCT using 3-arm group design at single center maternity unite/34–39 weeks’ pregnant women/*n* = 450/100% of participants completed the study	Women were given self-hypnosis lessons for pain control during labour in 3 sessions (37–39 weeks) by a hypnotherapist. Placebo group (Gp2) was given relaxation audio CD	It was measured by one question (birth was a positive or negative experience?) during 6 weeks after birth, as a secondary outcome.	Positive birth experience [Gp1 *N* = 108 (72.5%), Gp2 *N* = 105 (75.5%),control *N* = 118 (81.9%)]/The birth experience was unaffected by the antenatal group hypnosis training in the third trimester
Werner et al. 2013 Denmark	RCT using 3-arm group design conducted in university hospital/pregnant women/*n* = 1222/ 97.7% of them were followed	Women attended self-hypnosis classes (three 1-h classes during the last weeks of pregnancy) for childbirth.	The validated WIJMA Delivery Experience Questionnaire was used in six weeks postpartum.	Wijma score was 42.9 in the hypnosis Gp, 47.2 in the relaxation Gp and 47.5 in the usual care (*P* = 0.01)/the positive effect of the self-hypnosis was seen on the childbirth experience.
Simavli et al. 2014 Turkey	RCT in an obstetric department/ primiparous labouring women/*n* = 161/ follow up rate was 87.5%	During the active phase of labour, the self-selected music was played for mother, and it was continued until the end of the third stage of labour.	Visual analog scale for satisfaction with childbirth experience was used in 2, 12 and 24 h postpartum.	Satisfaction rate was higher in the music therapy Gp than control Gp (*P* = 0.001)/using music during labour declines pain and improves birth satisfaction.
Intrapartum care with minimal intervention				
Hundley et al. 1997 UK	A pragmatic RCT in a maternity hospital/women booking for childbirth/*n* = 2844/ 86% of them completed the study	Women received the midwifery labour care in a special unit with a minimum intervention during the labour and birth.	Participants were asked to grade their childbirth experience on an ordinal scale from 0 to 10 (0 = thoroughly unsatisfactory), after they discharge home.	There was no difference in Satisfaction with the birth experience among women in the midwives’ unit and routine labour care (*P* = 1.00).
McNiven et al. 1998 Canada	RCT in a teaching hospital/nulliparous women felt they were in early labour/*n* = 209/follow up rate was 96%	In admit of labour women received the early labour assessment; if they were not found to be in active phase, special support and practical advices (when they should be returned to the hospital) were given to them.	The validated LAS (Labour agentry scale) questionnaire was used in the postpartum period.	LAS score in early labour assessment = 158 versus in direct admission = 142 (*P* = 0.001)/ early labour assessment had a potential to improve women’s perception of their birth experience.
Spiby et al. 2008 UK	Pragmatic multi-central RCT/ women felt they were in early labour/*n* = 3514/ 75% of them completed the study but the main outcome (C/S rate) was measured from more than 85% of subjects	If woman felt she might be in early labour, a community midwife did a home visit to assess labour progress, provide support with coping strategies and guide on when to go to the hospital.	Experience of labour was measured by an invalid questionnaire which included several questions with five-point Likert scales, at 6 weeks following the birth.	Significant difference in mean score of questionnaire (2.02 versus 2.16 *P* = 0.0001) was seen between the home early labour assess and routine hospital admit/more positive birth experiences was seen in the home arm.
McLachan et al. 2016 Australia	RCT in tertiary care hospital/ pregnant women/ *n* = 2314/ completion rate was 88%	Women received a caseload midwifery care during the pregnancy, birth and postpartum.	Invalid questionnaire included several questions with Likert-scale response categories. It was used 2 months after birth.	Women in the caseload group reported more positive birth experience (adjusted OR 1.50, 95% CI 1.22–1.84)/ caseload midwifery have a positive effect on maternal birth memories.
Birth preparedness and readiness for complications				
Miamburg et al. 2010 Denmark	An RCT in a university hospital/ 10–22 weeks’ pregnant women were recruited/ *n* = 1193 / 97% of participants completed the study	Women received “ready for Child” training program in three sessions between 30 and 35 weeks of gestation. The program included details about labour onset and process, pain relief, how to overcome childbirth fear, care for newborn and transition to parenthood.	It was measured by a five-point Likert scale in 6 weeks and again 1 year and 5 year after the birth.	No significant differences in birth experience at 6 weeks postpartum (P = 0.79), but Five years after birth 188 women (20.8%) selected a “bad or very bad birth experience” option while 601 mothers (67.0%) had a “good or very good birth experience”/ Antenatal training declines unnecessary interventions in labour and improves long-term memories of maternal experience.
Kou et al. 2010 Taiwan	A cluster RCT in 7 hospitals/childbearing women/*n* = 330/ 90% of them were followed.	Each labouring woman had an individual “birth plan” which was designed by her obstetrician in prenatal visits. Mother was informed about the details of this plan and the labour care was according to the plan.	The validated childbirth experience questionnaire was used one day after the birth.	There was significant difference between the experimental Gp and the control Gp about childbirth experiences (*P* = 0.01)/Birth plan increased maternal positive birth experiences, and their control over the birth.

**Fig. 2:**
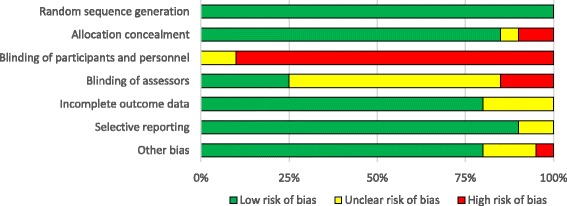
Authors’ judgments about risk of bias in included studies

The quality assessment of the included studies is shown in detail (Fig. 2). Methodological quality appraisal was conducted in two steps using two different tools, therefore, included studies were at low risk of bias in most of the items evaluated (except blinding). Given the interactive nature of the interventions, it was not possible to blind participants and blinding of the caregivers was difficult. Four of the included studies attempted to blind the caregivers, however they were not completely successful [38–41]. Eight of the 20 trials reported blinding status of the assessors; five of them were assessor-blinded [31, 35, 36, 39, 41] and three were not [30, 34, 42]. Most of the included studies concluded that their results were not influenced by the lack of blinding; however, they may have been impacted by the Hawthorne effect. The design of one of the studies increased the likelihood of “recall bias” [28]; this is considered as “other bias” in Fig. 2.

Almost 22,800 women took part in these trials. All 31 Turkish and 108 Persian articles were removed in the initial selection or quality assessment process; so, the final 20 trials were only in English. Four studies were carried out in the United Kingdom, three in Australia, three in the United States, two in Denmark, two in Canada and the reminder in seven other countries. Three trials did not state whether they were ethically approved by relevant institutional review boards. In this case, the authors were contacted by email and they confirmed that ethical approvals had been obtained prior to the implementation of interventions. From the 20 included trials, four were pregnancy interventions [38–40, 42], thirteen were birth interventions [27, 29–34, 36, 37, 41, 43–45] and three were the intervention of continuous care from pregnancy to birth [28, 35, 46]. The childbirth experience was measured as a primary outcome in seven studies [27, 29, 33, 40, 41, 44, 45] and as a secondary outcome in the remaining studies. Eight of the included studies used validated and reliable questionnaires for the measurement of the childbirth experience [28, 30, 33, 35, 37, 38, 42, 45]. Two studies measured the maternal experience by a visual analog scale [36, 41] and ten studies used Likert-type questions. Studies that used summated scales of the overall childbirth experience were not excluded because perception of overall experience was influenced by important factors, such as maternal expectations of birth, experienced complications during childbirth and the use of pain relief [47]. Most of the studies included a follow-up measurement after six weeks or less.

Other outcomes which were measured in the 20 studies included labour characteristics and birth outcomes, neonatal outcomes, labour pain, feelings of control during labour, ability to cope with fear, maternal self-esteem and self-worth, the rate of cesarean sections, the rate of epidural analgesia, satisfaction with care, post-partum depression and the success rate of breastfeeding. Neonatal outcomes were measured by various variables including birth weight [27, 33, 35, 36, 38, 40], Apgar score [27, 30, 31, 33–37, 39, 40, 43], meconium stained liquor [31, 37, 39, 43], admission to the NICU (neonatal intensive care unit) [27, 30, 34, 36, 37, 39, 42, 43, 46], resuscitation [30, 33, 34, 36], asphyxia [30], biochemical status [40], immediate mother-infant contact following birth [27], stillbirth/neonatal death [30, 34, 44], major congenital abnormality [30], and presence of complications [35, 36]. These neonatal outcomes did not associate with the childbirth experience in most studies, except one [46]. This study concluded that neonatal transfer to the NICU was associated with the negative childbirth experience.

Interventions designed to improve the childbirth experience can be categorized into four main groups as follows:

### Supporting women during birth

Seven studies compared continuous support versus usual care. Support was provided by a member of women’s social network (four studies) [27, 28, 32, 35], a nurse (one study) [30] or a hired companion (two studies) [29, 31]; all of them were trained to provide labour support. A certificated trainer had trained the birth companions in a single 2-h session [28, 35], multiple sessions [29–31] or via educational leaflets [27, 32]. The educational content of the training was almost the same and included information regarding the responsibilities of the birth companions, some knowledge of the labour progress and methods of providing continuous support. The control group of all seven trials received conventional care that did not involve continuous labour support.

**Fig. 3:**
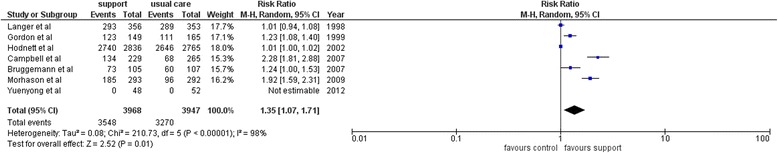
Comparison of positive perception of childbirth experience (dichotomous) between support and usual care

**Fig. 4:**
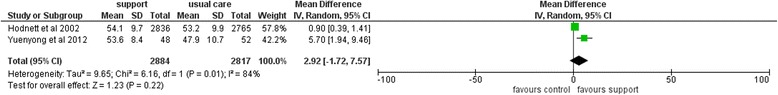
Comparison of birth experience scores (continuous) between support and usual care

Meta-analysis of six studies showed that women with companions were more likely to have a positive childbirth experience (RR = 1.35, 95% CIs: 1.07 to 1.71, *P* = 0.01) (Fig. 3). To request the essential data, the corresponding authors of three studies [30, 32, 35] were contacted by email. A study conducted by Yuenyong S. et al. (2012) did not divide women’s experiences into positive and negative [35]; the primary author was contacted and she confirmed that they did not collect dichotomous data on the childbirth experience. Therefore, continuous data of Yuenyong’s study [35] and the only homogeneous study [30] were pooled separately from the other six trials. Analysis of these two studies, did not show any significant difference in the birth experience (measured by Labour Agentry Scale [LAS]) among various groups (MD = 2.92, 95%CIs: − 1.72 to 7.57, *P* = 0.22) (Fig. 4).

In summary, meta-analysis of six studies indicates that supporting women during birth is an effective intervention for creating a more positive childbirth experience.

### Relaxation and pain relief during birth

**Fig. 5:**
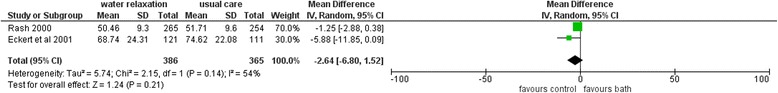
Comparison of birth experience scores (continuous) between water relaxation and usual care

Seven randomized controlled trials tested relaxation and pain relief strategies to promote a positive maternal childbirth experience. Two trials compared water immersion during labour with routine care [36, 37], two studies evaluated the effect of self-hypnobirthing [38, 39], one study compared three arms, which were massaging during labour, placebo relaxing music and usual care [42]. One study tested the effect of self-selected music during labour [41] and one study used epidural analgesia as a pain relief method for childbearing women [43]. As shown in Fig. 5, meta-analysis of the two similar studies involving immersion in a bath during labour found no difference between the childbirth experience in the intervention and control groups (MD = − 2.64, 95% CIs: − 6.80to 1.52, *P* = 0.21). According to the reports, participants in the control groups of both studies received relatively similar intrapartum care, which was provided by the nurse or midwife.

Hypnobirthing studies did not follow the same design of intervention and outcome measurements; therefore, their combination was impossible and a narrative synthesis of them is presented. One study assessed maternal perception of the birth experience by one dual-mode question; no difference was found between three groups (hypnobirthing, placebo music relaxation and usual care) regarding the positive childbirth experience (RR = 0.89, 95% CIs:0.78 to 1.00, *P* = 0.06). No statistical difference was evident in other aspects of the maternal experience such as satisfaction with the birth experience (*p* = 0.45), ability to cope with birth and sense of control during labour [39]. The second study assessed the birth experience by Wijma delivery experience questionnaire. Self-hypnobirthing was found as an effective method to improve the birth experience (Wijma score was 42.9 in the hypnobirthing group, 47.2 in the placebo relaxation techniques group and 47.5 in the usual care group, *P* = 0.01) [38]. The results of these two later studies seem to contradict each other.

One study addressed the effect of massaging by the mother or her birth companion on self-reported birth experience; more positive perceptions of labour and sense of control were detected in the intervention group versus usual care (MD = − 6.10, 95% CIs: − 11.49 to − 0.71, *P* = 0.03) [42]. The satisfaction score of mothers in the group receiving music therapy during labour was significantly higher than the control group at 2, 12 and 24 h after birth. (MD = 2.74, 95% CIs: 2.59 to 2.89, *P* < 0.001) [41]. In one study early initiation of the epidural analgesia with 10 ml 0.25% bupivacaine was used and it was followed with top-ups of 5–10 ml 0.25% bupivacaine, as requested by the woman; Results demonstrated that pain relief by epidural analgesia during labour had no impact on early and late satisfaction with the birth experience (RR = 0.94, 95% CIs: 0.80 to 1.09, *P* < 0.40) [43].

In summary, three of seven studies indicate that relaxation during labour is effective for improving the childbirth experience.

### Intrapartum care with minimal intervention

**Fig. 6:**
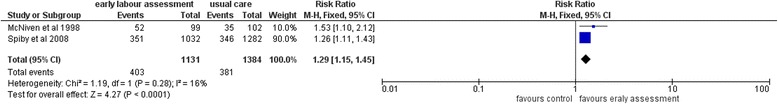
Comparison of satisfaction with birth experience (dichotomous) between early labour assessment and usual care

In order to reduce medical interventions, two studies conducted an early labour assessment [one in home [34] and one in hospital [33]]; women not found to be in active labour were educated on the signs of true labour and when to go to the hospital, given encouragement, and provided relaxation as a form of support. Both studies had the same approach for the control group; they were directly admitted to the labour unit which was managed by midwives. Analysis showed that women in the early labour assessment arm were more satisfied with their birth experience compared with those who were directly admitted to the hospital (RR = 1.29, 95% CIs:1.15 to 1.45, *P* < 0.001) (Fig. 6).

The study that compared a caseload midwifery care approach (prenatal, labour and postnatal care by a primary caseload midwife) with standard care found that participants in the intervention arm were more likely to report positive childbirth experience (RR = 1.14, 95% CIs:1.05 to 1.21, *P* < 0.001) [46]. In another related trial, labouring women were cared for in a special midwives’ unit with minimal medical intervention. Satisfaction with the birth experience did not significantly differ between the midwives’ unit and the usual labour ward (MD = 0.00, 95% CIs:-0.16 to 0.16, *P* = 1.00) [44].

In summary, three of four studies indicate that intrapartum care with minimal intervention is effective for creating a more positive birth experience.

### Birth preparedness and readiness for complications

Results of the “Ready for Child program” showed that women who had attended the antenatal birth classes reported a more favourable childbirth experience than the control group at evaluation five years after the birth (RR = 1.25, 95% CIs: 1.14 to 1.36, *P* < 0.001), however no difference was seen between the two groups at six weeks postpartum (RR = 0.99, 95% CIs: 0.93 to 1.06, *P* = 0.79) [40]. Another study used an individual birth plan to prepare women for birth and assessed the childbirth experience one day after delivery. There was a significant difference between the experimental and control groups in maternal birth experience (MD = 3.27, 95% CIs: 0.66 to 5.88, *P* = 0.01), feelings of control and fulfillment of expectations [45].

In summary, both studies indicate that birth preparedness is a successful strategy for improving the experience of birth.

## Discussion

### Principal findings

The study examined the effectiveness of women-based interventions on new mothers’ perception of the childbirth experience. The results of this systematic review identified four main categories of strategies applied in studies designed to improve the childbirth experience; some of these strategies have succeeded, while others have not.

### Summary of results and Comparison with other studies

The meta-analysis of trials assessing labour support revealed the positive effect of trained companion’s presence on maternal birth experience. While training approaches varied across studies, the common point of these interventions is physical presence and emotional support from the companion. Results showed that labour support from a person in a close relationship with the childbearing woman rather than a hired companion was more effective in the promotion of a positive birth experience. All of these findings are in keeping with the review on continuous support during childbirth [16]. In contradiction to these results, an analysis on LAS scores found no difference between support and control arms. This may be caused by two factors: firstly, the LAS score reflects only one aspect of the birth experience (experienced control during birth) and secondly, in one of the two relevant studies, the birth support was provided by staff nurses [30].

Evidence from RCTs evaluating the effect of pain relief methods during labour on maternal experience are conflicting. Water relaxation was not found as a useful technique to minimize the risk of a negative birth experience, neither was epidural analgesia. A previous review in 2002 confirmed that pain relief is not considered to be an effective variable in satisfaction with the childbirth experience [14]. Controversies in findings about the effect of self-hypnosis’s on the birth experience is probably due to the use of different measurement tools for childbirth experience; Cyna et al. measured experience as the number of mothers who felt their birth was a positive experience [39] while another study quantitatively assessed various aspects of the childbirth experience using a reliable and valid instrument [38]. Since the results of these two existing hypnobirthing trials are contradictory, it is not easy to determine whether the hypnosis was associated with more positive experiences of birth. It seems that there is a need to conduct a more adequately powered high quality hypnobirthing trial before this relaxation method can be considered as an effective strategy for preventing birth trauma.

According to the current evidence, among all labour relaxation techniques, only massage and music can be recommended clinically for improving women’s childbirth experiences. Social support involved in the massage intervention, casts doubt on the association between the massage component of the intervention and a positive childbirth experience. This means that a more positive childbirth experience might be the consequence of receiving support during massage rather than the massage itself [42]. The relaxation induced by self-selected music has been considered as a satisfying element in the listener’s experience [41].

Trials that aimed to prevent unnecessary obstetrical interventions through continuity of care from a primary midwife and empowering women with information to recognize the right time to go to hospital for the labour, were successful in promoting a positive maternal experience. Continuity of midwife care, improves the birth experience through various agents including maternal self-management of pain, ability to cope with the labour challenge, control over the birth process, support from trusted experts and reduction of stressful examinations and interventions [46, 48]. Intrapartum care in a specific home-like environment unit in hospital did not impact women’s childbirth experience [44]. Birth plans and preparedness for what would happen in the labour were identified as successful strategies for a better birth experiences. In this category, childbirth classes which were held two months before birth, were not found effective in the promotion of a positive birth experience.

In an effort to prevent negative and traumatic birth experiences, previous studies made recommendations to reduce related risk factors including inadequate labor support, high obstetric intervention rates, the occurrence of emergency cesarean section or instrumental delivery, and feelings of loss of control [46, 49–51]. Consistently, the strategies found in this systematic review were designed to control the above-mentioned risk factors. A common point of almost all successful strategies is to provide support during labour; this support was the main component in some interventions and was found as a hidden factor in the others [33, 34, 42, 46]. This finding is compatible with previous evidence suggesting that childbearing women need increased emotional care to prevent a PBT [18]. Behavior of maternity personnel plays an important role in pregnancy and birth memories. Ethically it is not appropriate to conduct a RCT on professional behavior of caregivers [14]. Humanitarian behavior is a duty of the practitioner; therefore, it should not be considered as an additional strategy in maternity care [1]. As there is no evidence to defend postpartum interventions for the prevention of psychological trauma [18], there is no high-quality data available to support prenatal interventions for the prevention of PBT. According to the current findings, it is optimal to focus on intrapartum care strategies for the prevention of negative and traumatic experiences of childbirth. We also recommend conducting more high-quality prenatal researches in this area.

### Implications for clinical practice

Due to the nature of this review, studies were included based on their outcomes rather than their interventions. The childbirth experience was considered as a gold-standard outcome for relevant interventions but it should be noted that each included study measured various outcomes. Some studies were unsuccessful in reducing the negative birth experience however had a positive impact on some other outcomes (labour pain, satisfaction with care, C/S rates, etc.) [37, 43, 44]. An analysis of each reported outcome is beyond the scope of this review.

The comparison of included interventions is a difficult task due to several factors including diversity in settings, socioeconomic disparities, variation in culture, and heterogeneity in outcome measurement tools. The socioeconomic variable was measured and controlled in 17 studies; they reported that socioeconomic status did not significantly differ between experiment and control groups. Other three studies did not assess participants’ socioeconomic status [28, 37, 43]. It should be noted access to maternity care is directly influenced by socioeconomic disparities [52]; this confounding factor has been compared between groups across different studies and no differences were detected. Independently, “access to prenatal care” was reported in some studies and its baseline similarity was confirmed between the control and experimental groups [16, 27, 31]. These findings show that the negative impact of socioeconomic disparities has been limited, as much as possible.

The quality of evidence about childbirth experience (outcome) is inconsistent across the studies because different tools and timings of administration had been used in trials that met the inclusion criteria. Childbirth experience is a multidimensional variable, so unidimensionality of its measurement scale might damage the quality of findings [16], as was seen in some included studies [30, 33, 35, 37, 42, 44]. The uncertainty of optimum time to assess birth experience is the main reason for heterogeneity in timing of outcome assessment [14]. All included studies measured the childbirth experience within three months of the birth; three studies repeated measurements after several months [36, 40, 43]. This finding is favourable because the first six months of birth is considered as the best time for surveys about maternity care used for health planning [44]. Similar future trials are needed to assess childbirth experience by a comprehensive and valid scale during a six-month postpartum period. According to current knowledge, the Childbirth Experience Questionnaire (CEQ) is the most comprehensive tool that covers many aspects of the maternal childbirth experience [52]. Researchers should validate CEQ before its use.

### Strengths and limitations of study

The authors of the current study attempted to include high-quality relevant trials as much as possible. The inclusion of studies from low, middle and high income countries with a considerable sample size is strength of this review. Participants in the control groups of all included studies received conventional care. The difference of the standard maternity care across cultural and geographic boundaries is an inherent limitation of this review. We carried out the necessary investigation about conventional care of studies that have been pooled in the meta-analyses. As mentioned in the results section, the routine care of homogenous trials was relatively similar. The conventional care in the labour support studies consisted relatively similar components including active management of labour, clinical assessment by obstetricians and midwives, and analgesia, as needed. The conventional care of water relaxation studies included midwife provided labour care, one-to-one support by nurse or midwife, and parenteral analgesia, as necessary. In early labour assessment studies, participants allocated to standard care did not received any instruction or advice related to the labour.

The lack of blinding in the included trials is an unavoidable limitation in this review. To reduce response bias, some studies had used blinded interviewers and all studies had applied self-administrated method. Although self-evaluation measures might limit the accuracy of the findings, they are the most direct and valid approach currently available to determine the subjects’ perception of experience [53]. To have homogeneous studies, the target population in the selected trials were low-risk pregnant women; as these samples do not represent the total population of pregnant women, it can be considered as a limitation of this review.

### Directions for future studies

Future researches aiming to improve the birth experience should evaluate psychological birth trauma as an outcome of their interventions. Interventions to help practitioners learn more about mother-friendly birth should be designed and assessed. In addition, it would be useful to assess the effectiveness of partner-based interventions on the maternal childbirth experience. One of the most important sub-groups requiring evidence to inform practice, is women with a fear of childbirth. Therefore, interventions designed for this special sub-group would deserve a separate review.

## Conclusion

This systematic review provides a summary of available strategies that had been designed to improve the maternal experience of childbirth. The aim of this study was to classify these strategies and identify those that were successful. Four main categories of strategies are supporting childbearing women, relaxation and pain relief during birth, minimizing obstetric interventions, and birth preparedness. Successful interventions were supporting women during labour, relaxation through massage and music, early labour assessment to minimize obstetric interventions, and birth preparedness. The main recommendation of this review is that emotional support programs for childbearing women should be implemented in countries’ maternal health plans. These programs can comprise a combination of successful strategies such as continuous labour support by a familiar person, reassuring physical contact using massage, and the continuity of midwifery care. There is a need for more clinical strategies that result in positive childbirth experiences. The results of this study might be helpful in planning clinical approaches and designing future studies regarding the prevention of negative and traumatic birth experiences.

## Additional files

Additional file 1: Search strategy. (DOCX 17 kb)

Additional file 2: Modified version of the Cochrane Public Health Group Data Extraction Template. (DOCX 58 kb)
