# Supplementary material for: Creating a positive perception of childbirth experience: systematic review and meta-analysis of prenatal and intrapartum interventions
Source: Reprod Health. 2018 May 2;15:73. doi: 10.1186/s12978-018-0511-x (PMC5932889; doi:10.1186/s12978-018-0511-x)
Supplement: Supplementary file 1 — Search strategy. (DOCX 17 kb) [file 12978_2018_511_MOESM1_ESM.docx]

Additional file 1: Search strategy

| Database | Key words’ combination |
| --- | --- |
| Embase  Medline  Scopus | #1 “childbirth experience*”.mp. OR “experience of childbirth*”.mp.  #2 “birth experience*”.mp. OR “experience of birth*”.mp.  #3 “labour experience*”.mp. OR “experience of labour*”.mp.  #4 “delivery experience*”.mp. OR “experience of delivery*”.mp.  #5 “maternal experience*”.mp. OR “experience of mother*”.mp. OR “mother’s experience”.mp.  #6 birth perception*”.mp. OR “perception of birth*”.mp.  #7 “childbirth perception*”.mp. OR “perception of childbirth*”.mp.  #8 OR/#1 - #7  #9 positive*.mp.  #10 negative*.mp.  #11 traumatic*.mp.  #12 prenatal*.mp.  #13 intrapartum*.mp.  #14 OR/#9 - #13  #15 trial*.mp.  #16 RCT*.mp.  #17 intervention*.mp.  #18 prevention*.mp.  #19 OR/#16 - #18  #20 AND/#8, #14, #19 |
| Web of Sciences | (“childbirth experience*” OR “experience of childbirth*” OR “birth experience*” OR experience of birth*” OR “labour experience*” OR “experience of labour*” OR “delivery experience*” OR “experience of delivery*” OR “maternal experience*” OR “experience of mother*” OR “mother’s experience” OR “birth perception*” OR “perception of birth*” OR “childbirth perception*” OR “perception of childbirth*”) AND (positive* OR negative* OR traumatic* OR prenatal* OR intrapartum*) AND (trial* OR RCT* OR intervention* OR prevention*) |
| CENTRAL | #1 “childbirth experience*” OR “experience of childbirth*”  #2 “birth experience*” OR “experience of birth*”  #3 “labour experience*” OR “experience of labour*”  #4 “delivery experience*” OR “experience of delivery*”  #5 “maternal experience*” OR “experience of mother*”. OR “mother’s experience”  #6 birth perception*” OR “perception of birth*”.  #7 “childbirth perception*” OR “perception of childbirth*”  #8 OR/#1 - #7  #9 positive*  #10 negative*  #11 traumatic*  #12 prenatal*  #13 intrapartum*  #14 OR/#9 - #13  #15 AND/ #8, #14 |
