# Supplementary material for: Creating a positive perception of childbirth experience: systematic review and meta-analysis of prenatal and intrapartum interventions
Source: Reprod Health. 2018 May 2;15:73. doi: 10.1186/s12978-018-0511-x (PMC5932889; doi:10.1186/s12978-018-0511-x)
Supplement: Supplementary file 2 — Modified version of the Cochrane Public Health Group Data Extraction Template. (DOCX 58 kb) [file 12978_2018_511_MOESM2_ESM.docx]

| **Study ID:** | **Report ID :** | Date form completed: |
| --- | --- | --- |
| First author: | Year of study: | Data extractor: |
| Citation: | | |

**1. General Information**

| Publication type Journal Article ⬜ Abstract ⬜ Other (specify e.g. book chapter) | |
| --- | --- |
| Country of study: | |
| Funding source of study: | Potential conflict of interest from funding? |

**2. Study Eligibility**

| **Study Characteristics** | | | **Page/** |
| --- | --- | --- | --- |
| **Type of study**  (Review authors to add/remove designs based on criteria specified in protocol) | ⬜ Randomised Controlled Trial (RCT)  ⬜ Cluster Randomised Controlled Trial (cluster RCT) | |  |
|  | *Does the study design meet the criteria for inclusion?*  Yes⬜ No ⬜ 🡪**Exclude** Unclear ⬜ | |  |
|  | Description in text: | |  |
| **Participants**  (Review authors insert inclusion criteria as defined in Protocol) | Describe the participants included: | |  |
|  | Are participants defined as a group having specific social or cultural characteristics? | Yes ⬜ No ⬜ Unclear ⬜  Details: |  |
|  | How is the geographic boundary defined? | Details:  Specific location (e.g. state / country): |  |
|  | *Do the participants meet the criteria for inclusion?* | Yes ⬜ No ⬜ 🡪**Exclude** Unclear ⬜ |  |

**Intervention**

| **Types of intervention**  (Review authors insert inclusion criteria as defined in Protocol) | Strategies included in the intervention | |  | |  |
| --- | --- | --- | --- | --- | --- |
|  | Focus of the intervention | |  | |  |
|  | *Does the intervention meet the criteria for inclusion?* | | Yes ⬜ No ⬜ 🡪**Exclude** Unclear ⬜ | |  |
| **Duration of intervention** | Start date: | Stop date: | | Intervention duration: |  |
|  | *Is the duration of intervention adequate for inclusion?* | | Yes ⬜ No ⬜ 🡪**Exclude** Unclear ⬜ | |  |
| **Types of outcome measures**  (Review authors insert inclusion criteria as defined in Protocol) | List outcomes: | |  | |  |
|  | Outcome measured at a population level or individual level? | | Details: | |  |
|  | *Do the outcome measures meet the criteria for inclusion?* | | Yes ⬜ No ⬜ 🡪**Exclude** Unclear ⬜ | |  |

**Summary of Assessment for Inclusion**

| **Include in review** ⬜ **Exclude from review ⬜** | |
| --- | --- |
| Independently assessed, and then compared? Yes ⬜ No ⬜ | Differences resolved Yes ⬜ No ⬜ |
| Request further details? Yes ⬜ No ⬜ | Contact details of authors: |
| **Notes:** | |

DO NOT PROCEED IF PAPER EXCLUDED FROM REVIEW

**3. Study details**

| **Study intention** | **Descriptions as stated in the report/paper** | **Page/** |
| --- | --- | --- |
| Aim of intervention |  |  |
| Aim of study |  |  |
| Equity pointer: Social context of the study |  |  |
| Start and end date of the study |  |  |
| Total study duration |  |  |

| **Methods** | **Descriptions as stated in the report/paper** | **Page/** |
| --- | --- | --- |
| Method/s of recruitment of participants |  |  |
| Inclusion/exclusion criteria for participation in study |  |  |
| Representativeness of sample: Are participants in the study likely to be representative of the target population? |  |  |
| Total number of intervention groups |  |  |
| Assessment time of outcomes |  |  |
| Sample size calculation:  What assumptions were made?  Were these assumptions appropriate? |  |  |
| What was the unit of randomisation?  Allocation by individuals or cluster/groups |  |  |
| What was the unit of analysis?  **Is this the same as the unit of randomisation?** |  |  |
| Statistical methods used and appropriateness of these methods |  |  |

**Results**

| **Participants**  *Include if relevant* | **Include information for each group (i.e. intervention and controls) under study** | **Page/** |
| --- | --- | --- |
| 1. What percentage of selected individuals agreed to participate? |  |  |
| 1. Total number randomised (or total pop. at start of study for NRCTs) |  |  |
| 1. Number allocated to each intervention group (no. of individuals) |  |  |
| 1. For cluster trials, number of clusters, number of people per cluster |  |  |
| 1. Where there any significant baseline imbalances? | Yes ⬜ No ⬜ Unclear ⬜ Details: |  |
| 1. Number and reason for (and sociodemographic differences of) withdrawals and exclusions for each intervention group |  |  |
| 1. What percentage of patients completed the study? |  |  |
| 1. What percentage of participants received the allocated intervention or exposure of interest? |  |  |
| 1. Is the analysis performed by intervention allocation status (intention to treat) rather than the actual intervention received? Have any attempts been made to impute missing data? |  |  |
| 1. Age (median, mean and range if possible) |  |  |
| - Sociodemographics (eg. Educational level, literacy level, soci-economic status, first language. Also consider possible proxies for these e.g. low baseline nutritional status ) |  |  |

**Outcomes**

| **Question** | **Outcome: Childbirth experience** | **Maternal secondary outcomes** |
| --- | --- | --- |
| Is this dichotomous or continuous outcome? |  |  |
| Outcome definition (with diagnostic criteria if relevant) |  |  |
| Time points measured |  |  |
| Time points reported |  |  |
| Is there adequate latency for the outcome to be observed? |  |  |
| For scales – upper and lower limits and indicate whether high or low score is good |  |  |
| How is the measure applied? Telephone survey, mail survey, in person by trained assessor, routinely collected data, other |  |  |
| How is the outcome reported? Self or study assessor |  |  |
| Is this outcome/tool validated? |  |  |
| …And has it been used as validated? |  |  |
| Is it a reliable outcome measure? |  |  |
| Is there adequate power for this outcome? |  |  |
| final outcome |  |  |

**Other relevant information**

| Were outcomes relating to harms/unintended effects of the intervention described? |  |  |
| --- | --- | --- |
| Potential for author conflict |  |  |
| Key conclusions of the study authors |  |  |
| Could the inclusion of this study potentially bias the generalizability of the review? Equity pointer: Remember to consider whether disadvantaged populations may have been excluded from the study. |  | |
| Is there potential for differences in relative effects between advantaged and disadvantaged populations? |  | |
| Are interventions likely to be aimed at the disadvantaged? (e.g. school meals aimed at poor children). |  | |
| Issues affecting directness |  | |
| References to other relevant studies |  | |
| Additional notes by review authors |  | |
| Correspondence required for further study information (from whom, what and when) |  | |
